# Supplementary material for: RBL2 represses the transcriptional activity of Multicilin to inhibit multiciliogenesis
Source: Cell Death Dis. 2024 Jan 22;15(1):81. doi: 10.1038/s41419-024-06440-z (PMC10803754; doi:10.1038/s41419-024-06440-z)
Supplement: Supplementary file 1 — Original Data File [file 41419_2024_6440_MOESM1_ESM.pdf]

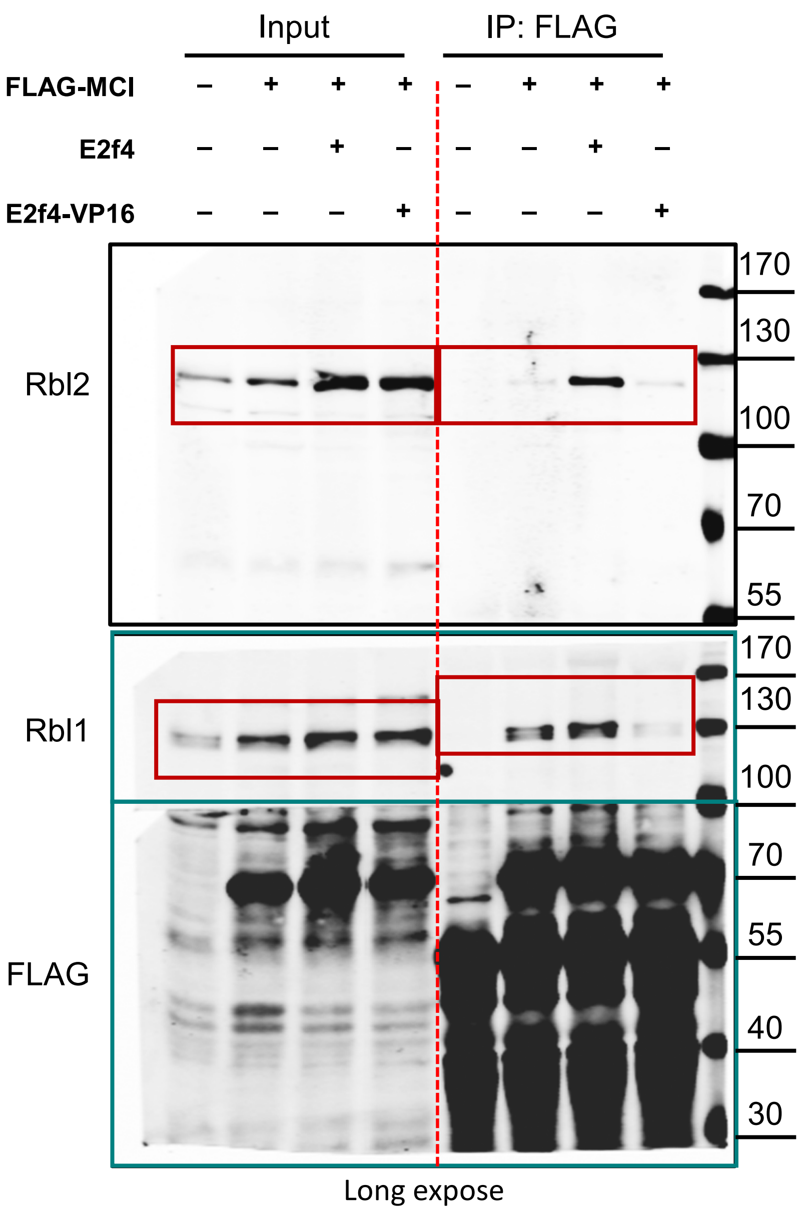


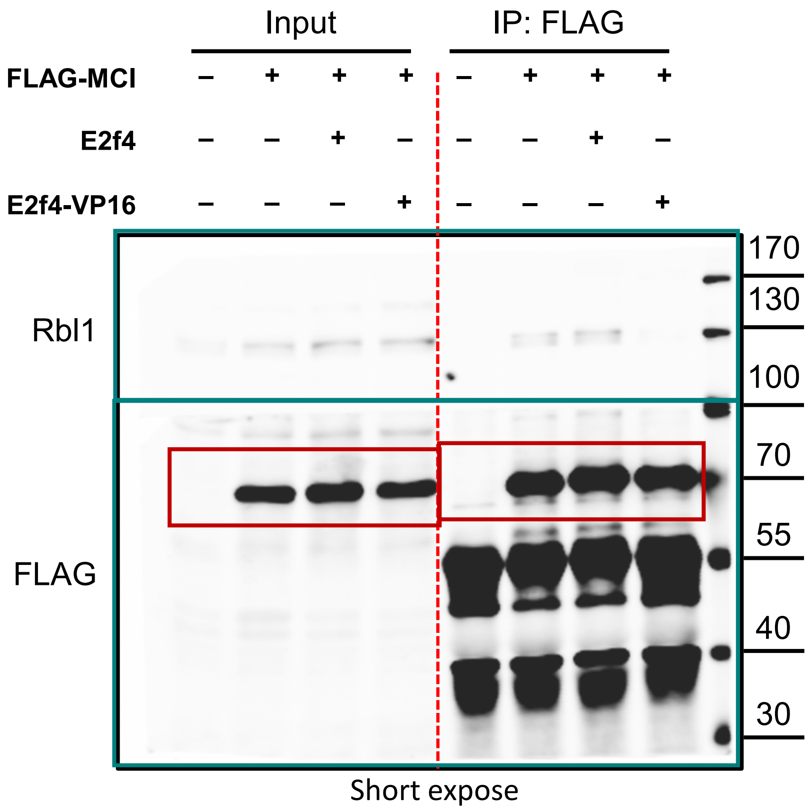


**Raw Data related to Figure 1**

Full immunoblots for figure 1D

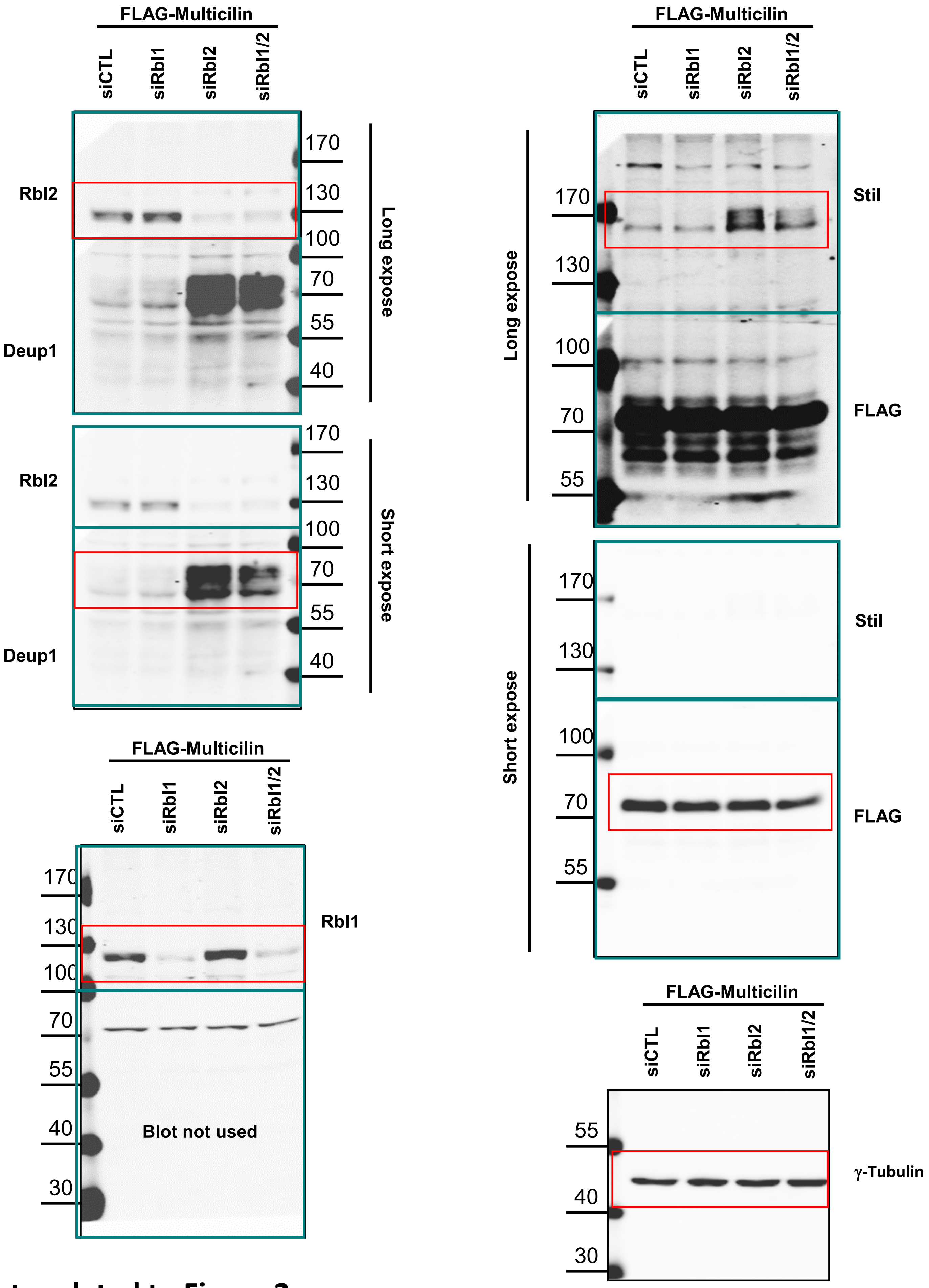


# Raw Data related to Figure 2a

Full immunoblots related to Figure 2a

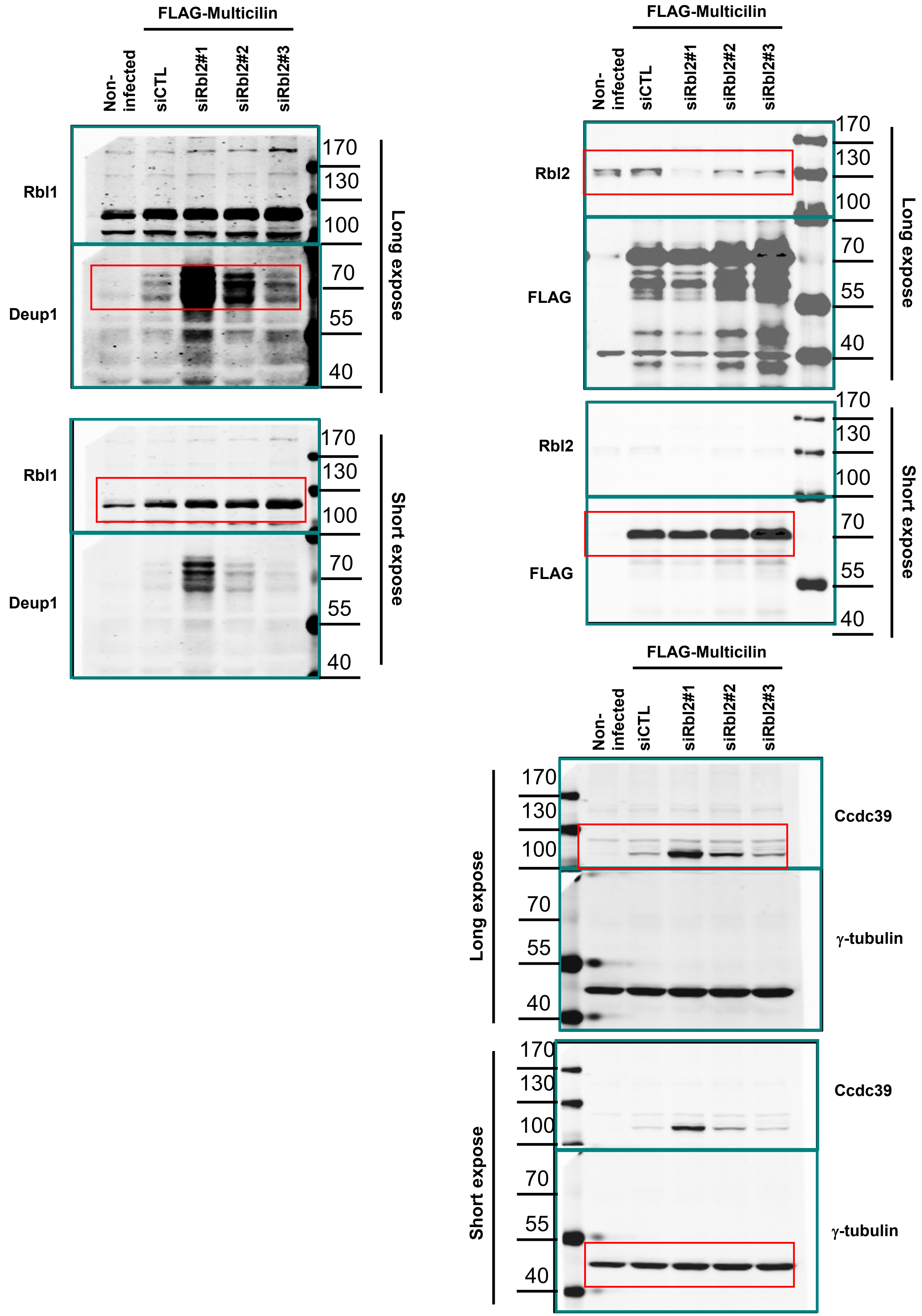


**Raw Data related to Figure 2b**

Full immunoblots related to **related to Figure 2b**

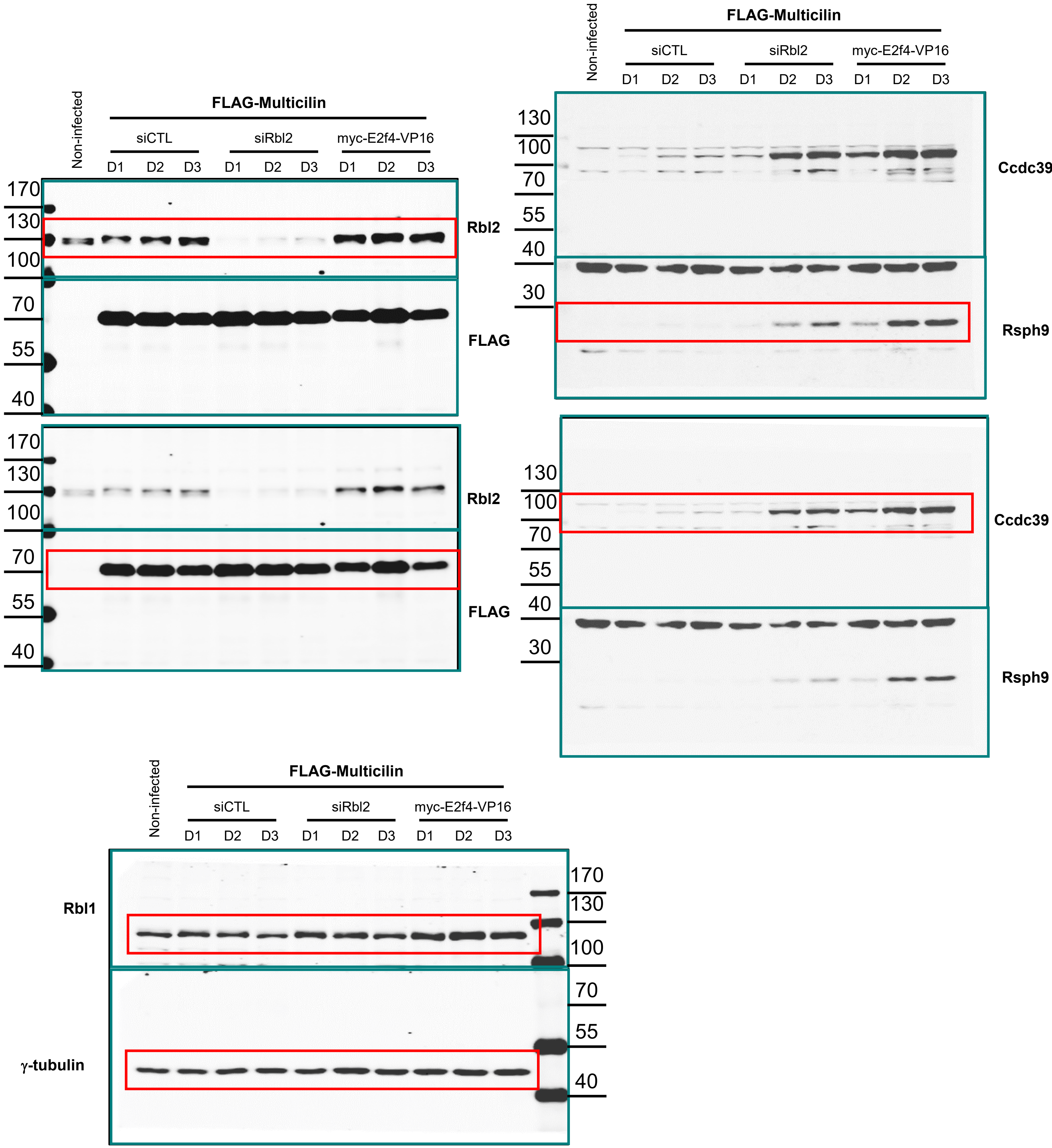


## Raw Data related to Figure 2d

Full immunoblots related to Figure 2d

shRBL2#1

RBL2

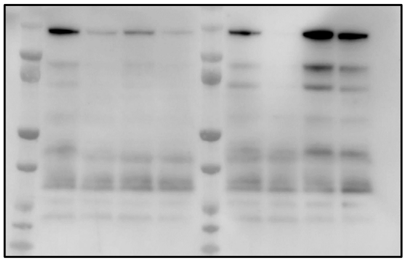

CCDC39

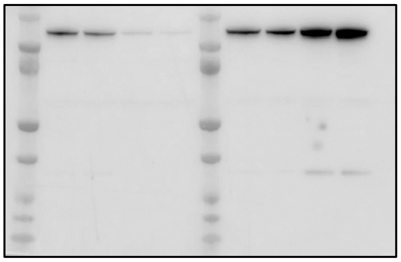

P73

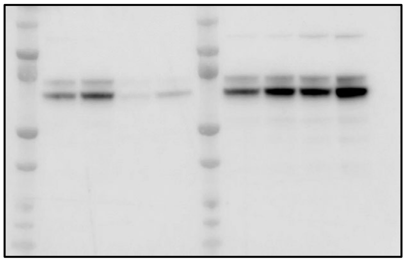

FOXJ1

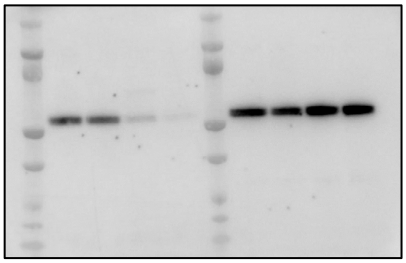

ß-ACTIN

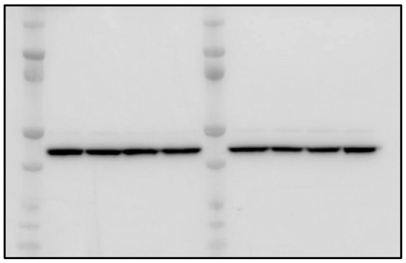

| Lane | 1 | 2 | 3 | 4 | 5 | 6 | 7 | 8 | 9 | 10 |
|---|---|---|---|---|---|---|---|---|---|---|
| pSico-mCh-Puro shRNA Target | - | NTC# #1 | RBL2 #1 | NTC# #1 | RBL2 #1 | - | NTC# #1 | RBL2 #1 | NTC# #1 | RBL2 #1 |
| HBEC Donor Number# | - | #1 | #1 | #2 | #2 | - | #3 | #3 | #4 | #4 |
| Protein Ladder (Bio-Rad #1610374) | + | - | - | - | - | + | - | - | - | - |

---

RBL2

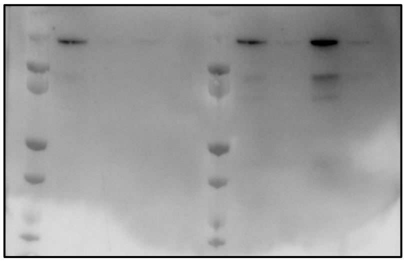

CCDC39

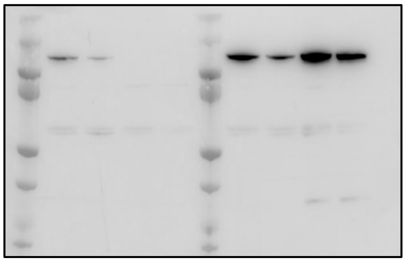

P73

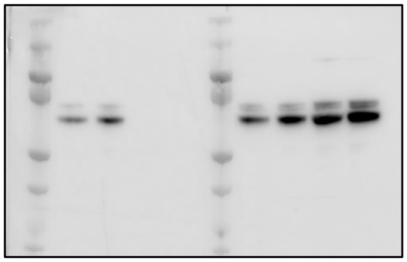

FOXJ1

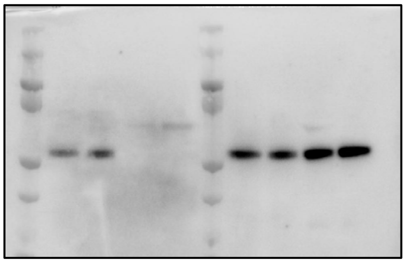

ß-ACTIN

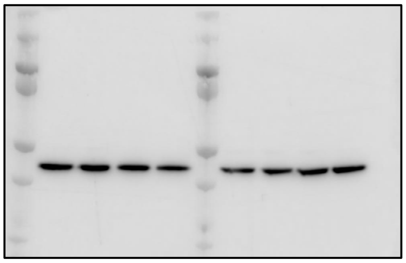

| Lane | 1 | 2 | 3 | 4 | 5 | 6 | 7 | 8 | 9 | 10 |
|---|---|---|---|---|---|---|---|---|---|---|
| pLKO.1 Target | - | NTC# #2 | RBL2 #2 | NTC# #2 | RBL2 #2 | - | NTC# #2 | RBL2 #2 | NTC# #2 | RBL2 #2 |
| HBEC Donor Number# | - | #1 | #1 | #2 | #2 | - | #3 | #3 | #4 | #4 |
| Protein Ladder (Bio-Rad #1610374) | + | - | - | - | - | + | - | - | - | - |

**Raw data related to figure Fig 4d and e.**

Full blots for 4d

Biological replicates for quantification in 4e

HBEC donor #2 was excluded from analysis due to failure to meet differentiation standards in the NTC control.

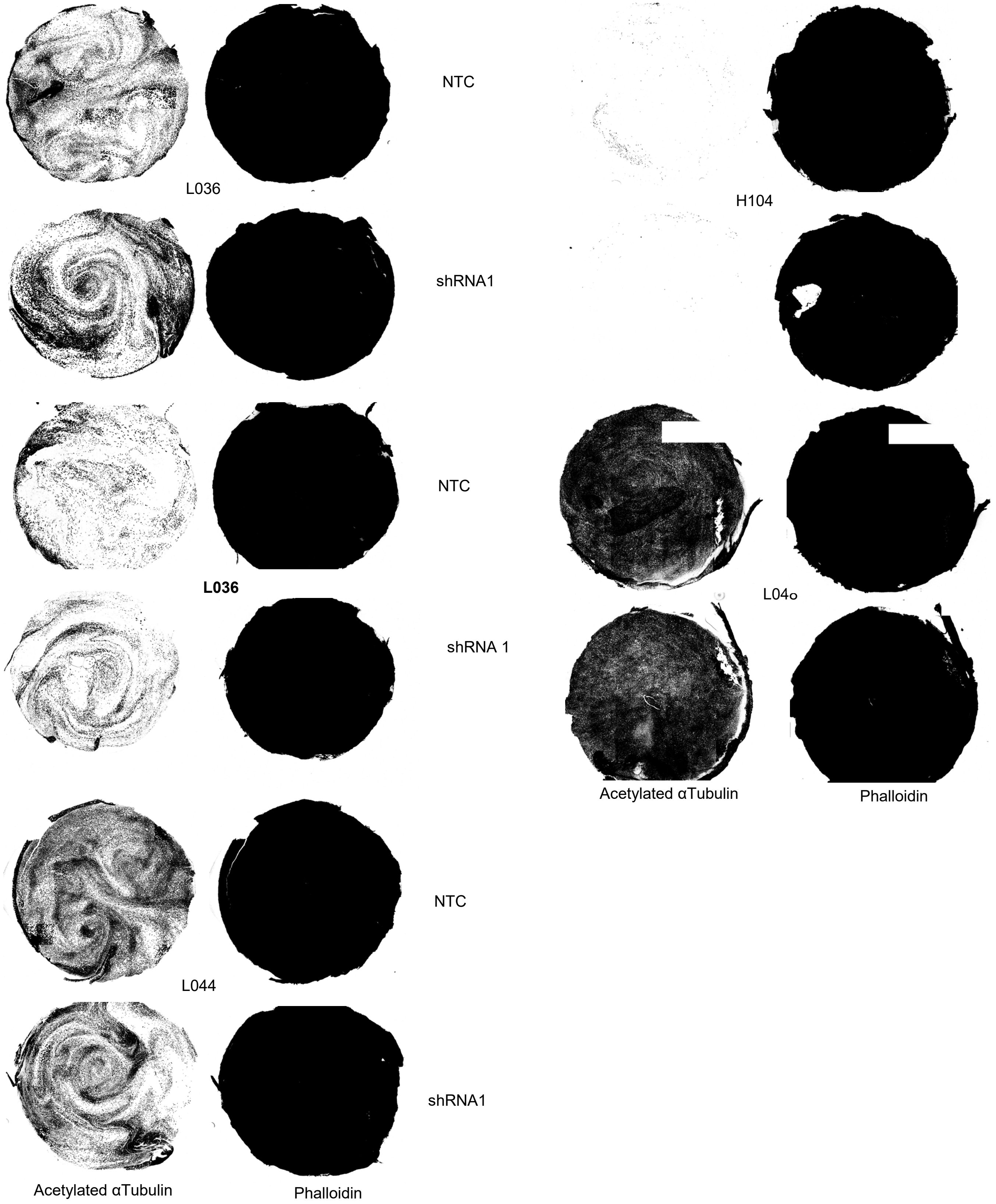


**Raw data related to figure Fig 4f.**

JPEGs of tile scans of 6.5mm transwell inserts with thresholds set for positive staining to quantitate percentage of apical cilia coverage area

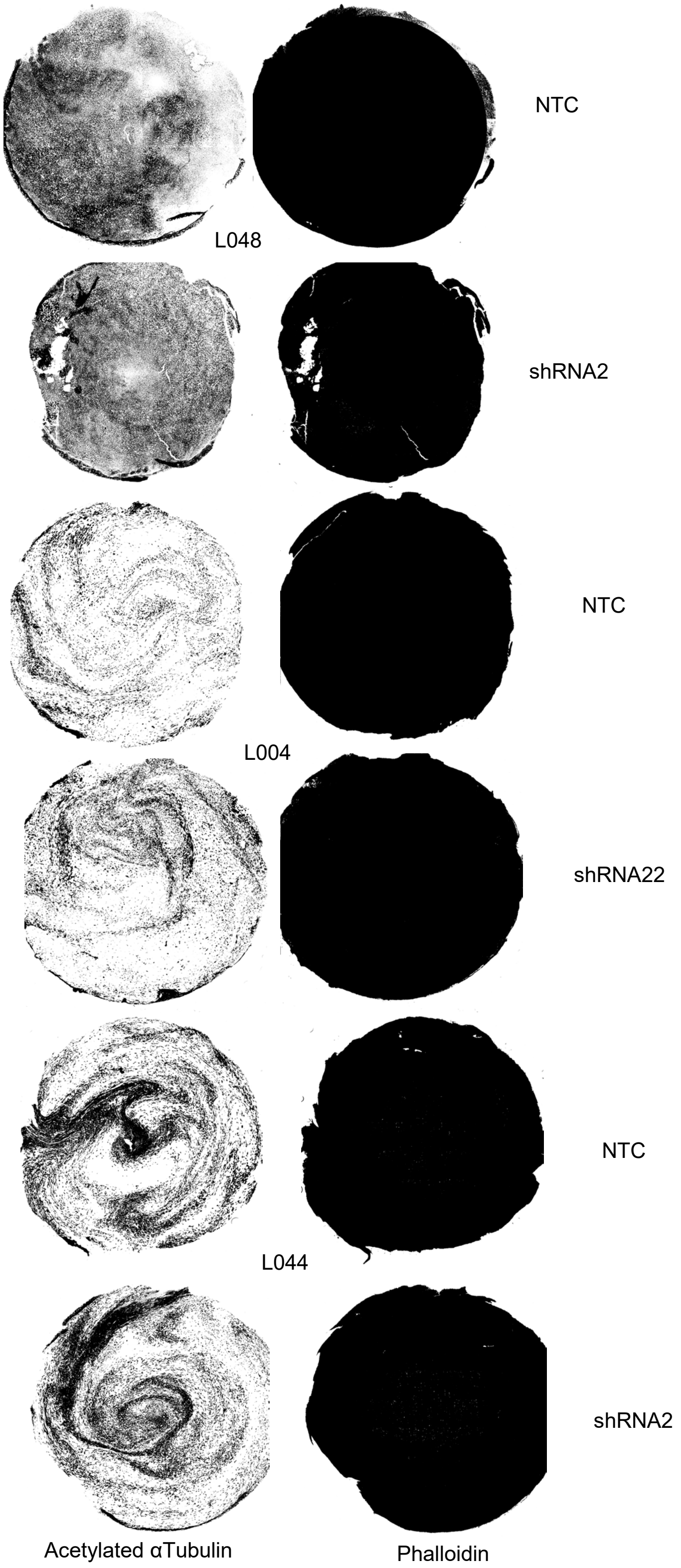


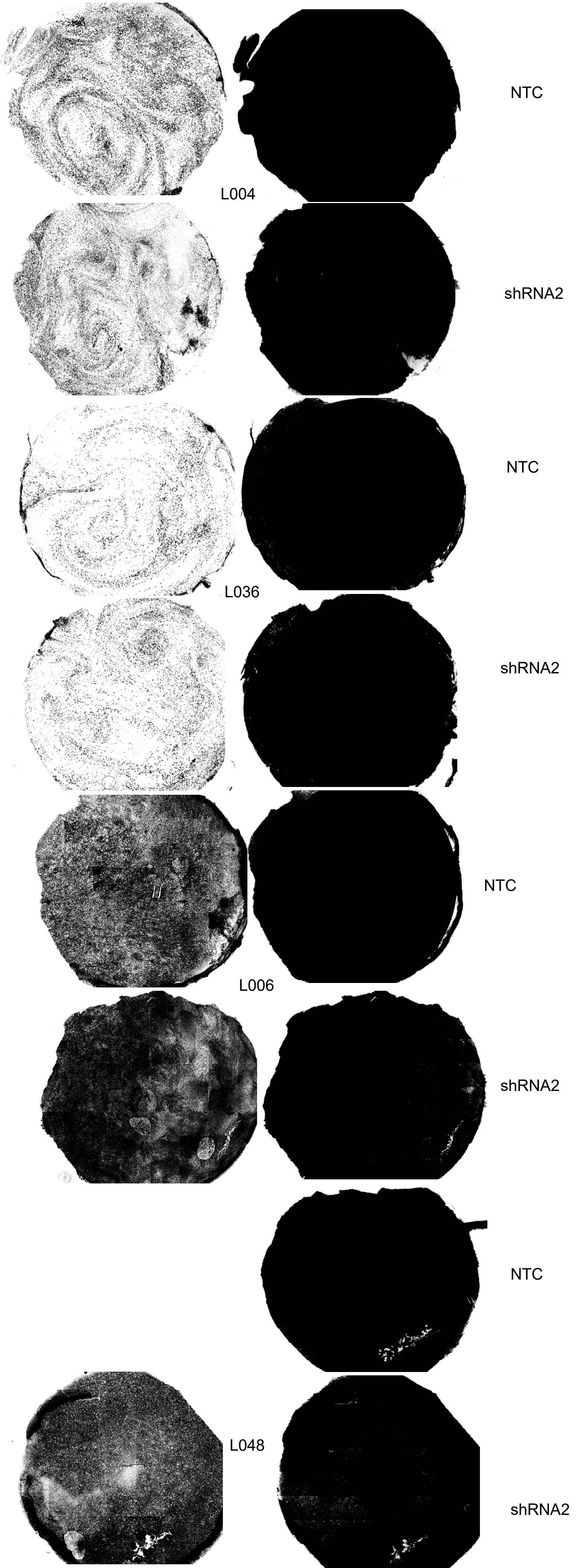


**Raw data related to figure Fig 4f.**
JPEGs of tile scans of 6.5mm transwell inserts with thresholds set for positive staining to quantitate percentage of apical cilia coverage area for Fig 4f.

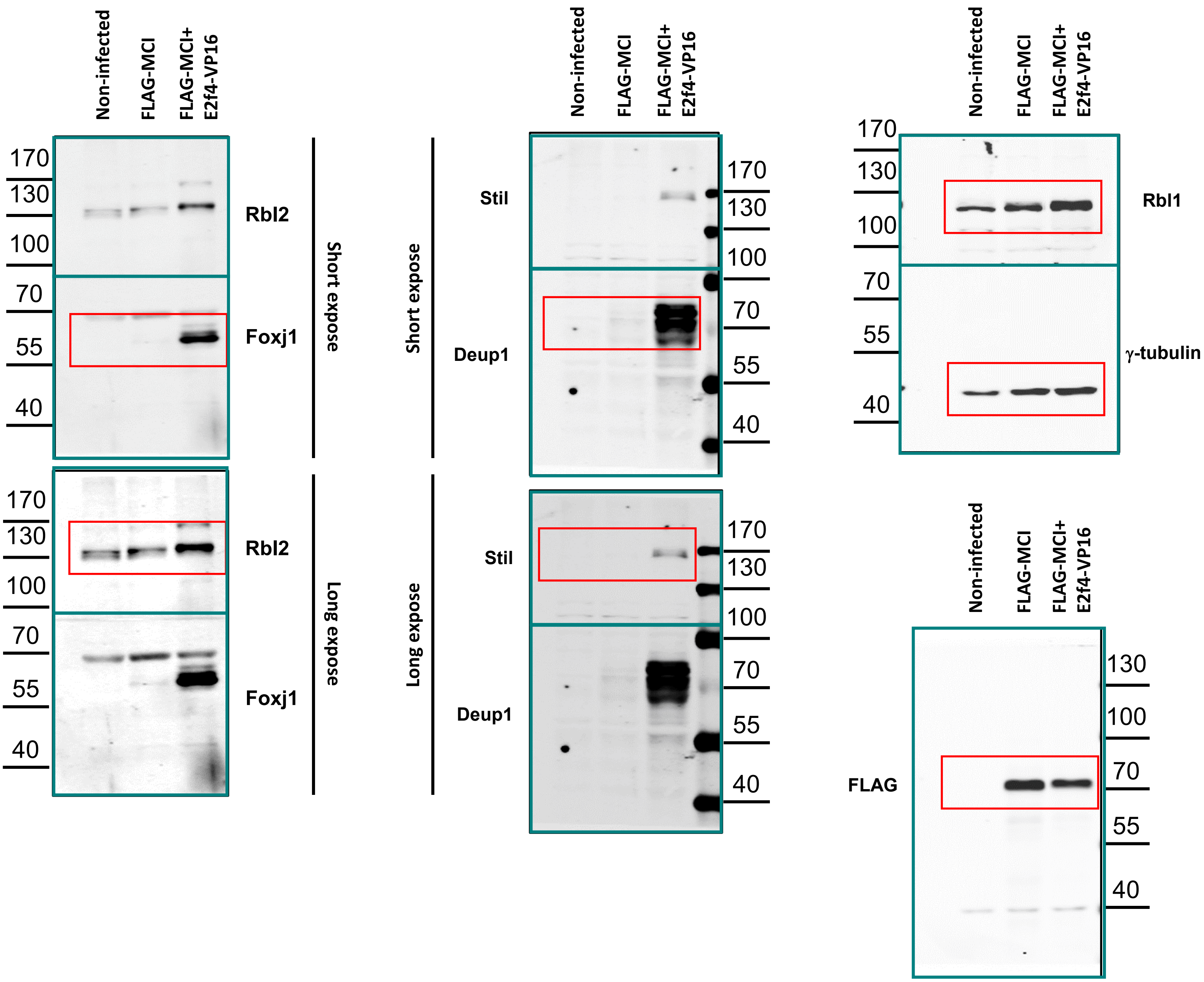


## Raw Data related to Supplementary Figure 1

Full immunoblots for Supplement 1B

Phospho-RBL2 S672

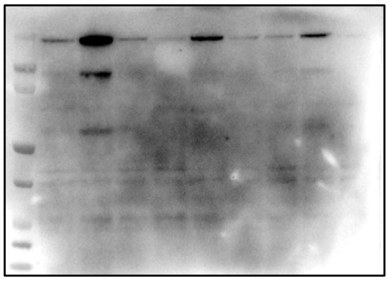

Total RBL2

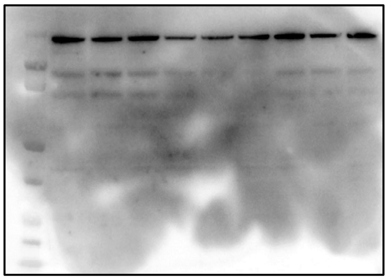

TP73

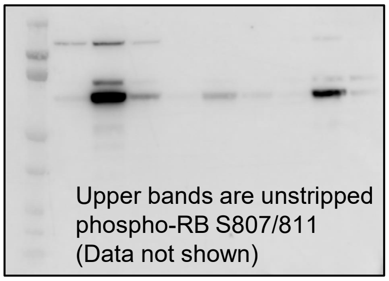


FOXJ1

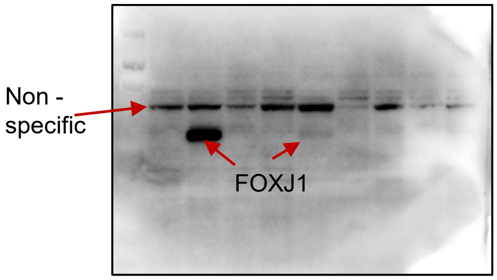


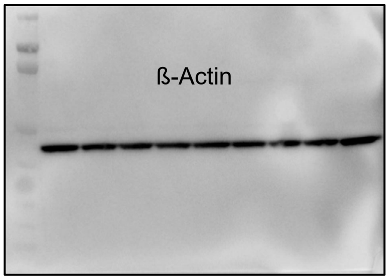


| Lane | 1 | 2 | 3 | 4 | 5 | 6 | 7 | 8 | 9 | 10 |
|---|---|---|---|---|---|---|---|---|---|---|
| Condition | - | Day 0 | Day 4 ALI | Day 4 Sub | Day 0 | Day 4 ALI | Day 4 Sub | Day 0 | Day 4 ALI | Day 4 Sub |
| HBEC Donor Number# | - | #1 | #1 | #1 | #2 | #2 | #2 | #3 | #3 | #3 |
| Protein Ladder (Bio-Rad #1610374) | + | - | - | - | - | - | - | - | - | - |

Non-specific bands were observed at higher exposure times needed for FOXJ immunoblots for HBECs at 4 days of differentiation.

---

Phospho-RBL2 S672

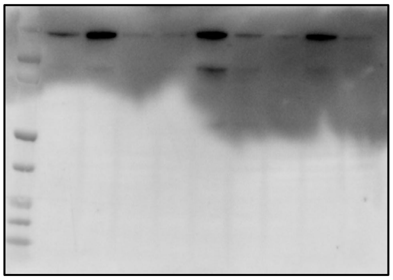

Total RBL2

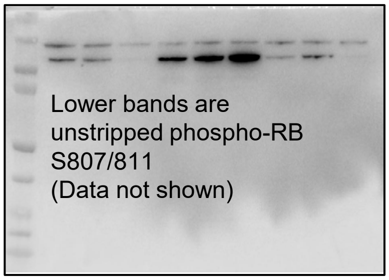


TP73

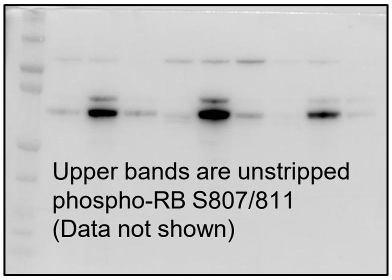


FOXJ1

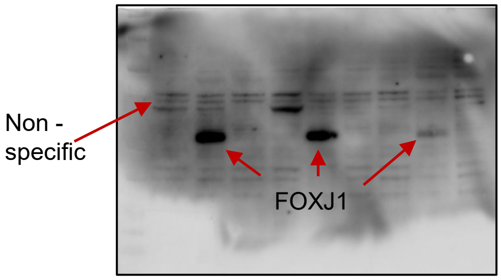


ß-Actin

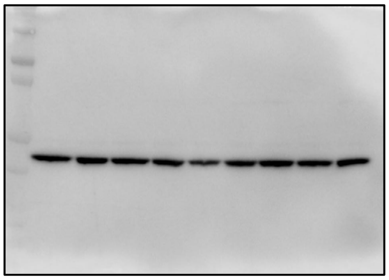

| Lane | 1 | 2 | 3 | 4 | 5 | 6 | 7 | 8 | 9 | 10 |
|---|---|---|---|---|---|---|---|---|---|---|
| Condition | - | Day 0 | Day 4 ALI | Day 4 Sub | Day 0 | Day 4 ALI | Day 4 Sub | Day 0 | Day 4 ALI | Day 4 Sub |
| HBEC Donor Number# | - | #4 | #4 | #4 | #5 | #5 | #5 | #6 | #6 | #6 |
| Protein Ladder (Bio-Rad #1610374) | + | - | - | - | - | - | - | - | - | - |

Non-specific bands were observed at higher exposure times needed for FOXJ immunoblots for HBECs at 4 days of differentiation.

# Raw Data related to Figure 5c and f

Full immunoblots for Figure 5C and biological replicates for quantification in Figure 5F .

RBL2

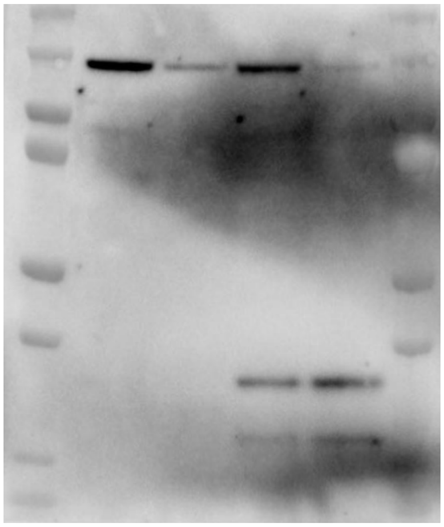

TP73

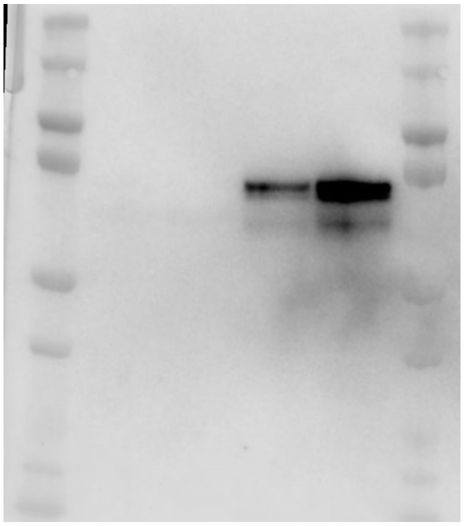

FLAG (Multicilin)

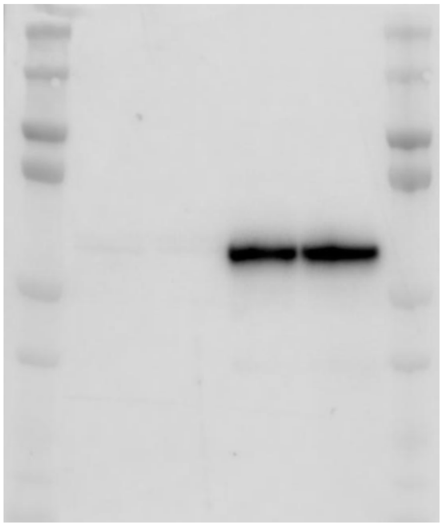

ß-actin

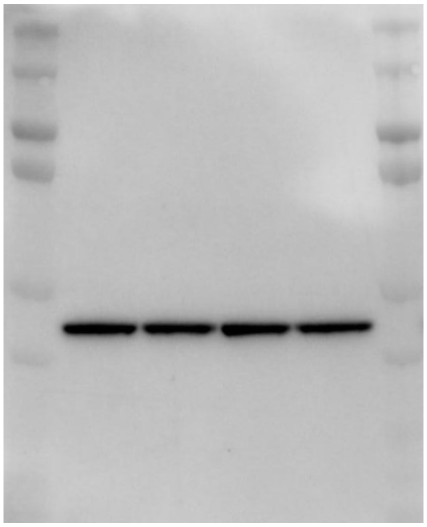

| Lane | 1 | 2 | 3 | 4 | 5 | 6 |
|---|---|---|---|---|---|---|
| Condition | - | Vehicle (H2O)/ shRNA NTC#2 | Vehicle (H2O)/ shRNA RBL2#2 | Doxycycline/ shRNA NTC#2 | Doxycycline shRNA RBL2#2 | - |
| HBEC Donor Number# | - | #5 | #5 | #5 | #5 | - |
| Protein Ladder (Bio-Rad #1610374) | + | - | - | - | - | + |

**Raw Data associated with Figure 6D**

Whole immunoblots for Figure 6D.
